# Editorial: Advances in pharmacotherapy for alcohol use disorder: from mechanisms to clinical interventions

**DOI:** 10.3389/fphar.2025.1671114

**Published:** 2025-08-15

**Authors:** Regina A. Mangieri, Sunil Sirohi, Ezequiel Marron Fernandez de Velasco, Seungwoo Kang

**Affiliations:** ^1^ Division of Pharmacology and Toxicology, College of Pharmacy, The University of Texas at Austin, Austin, TX, United States; ^2^ Laboratory of Endocrine and Neuropsychiatric Disorders, Division of Basic Pharmaceutical Sciences, College of Pharmacy, Xavier University of Louisiana, New Orleans, LA, United States; ^3^ Department of Pharmacology, University of Minnesota, Minneapolis, MN, United States; ^4^ Department of Pharmacology and Toxicology, Medical College of Georgia, Augusta University, Augusta, GA, United States

**Keywords:** alcohol use disorder (AUD), striatum, GLP-1, glucagon-like peptide-1, high fat diet (HFD), pharmacotherapeutics, traditional medicine

To overcome alcohol use disorder (AUD), current therapeutic strategies encompass behavioral, psychosocial, and medication-based approaches designed to reduce alcohol use and the associated comorbidities. However, current approved treatments fail many patients, which points to the unmet need to understand mechanisms underlying AUD and develop novel therapeutic targets addressing this disorder’s complex neurobiological foundations. This research collection on this topic aims to provide new information about brain adaptation mechanisms, molecular targets affected by alcohol exposure, pharmacological interventions for alcohol intake, and assessments of strategies for developing practically effective medications for AUD. The findings collectively illustrate diverse avenues for improving the outcomes of AUD interventions.

Pathophysiological adaptation in the striatum has been recognized as a principal brain dysfunction resulting from chronic alcohol exposure, specifically contributing to maladaptive reward-seeking behaviors (Corbit and Janak, 2016). Given the different roles of striatal subregions in shaping reward-seeking patterns and motivation, the detailed characterization of striatal adaptations to chronic alcohol consumption is important to understand the progression from voluntary to inflexible alcohol-seeking behaviors. Recent research by Duffus et al. employed mass spectrometry analysis of protein abundance in the dorsomedial, dorsolateral, and nucleus accumbens subregions of the striatum, at two different abstinence time points following chronic, voluntary alcohol drinking in male and female mice (Duffus et al.). Their comprehensive proteomics data illuminated brain adaptations, such as changes in neurodegeneration-associated proteins, that differed as a function of abstinence duration, subregion, and sex. A general feature, however, was that chronic alcohol drinking appeared to primarily alter proteins important for neuronal structure and cellular health, rather than induce neuroinflammation in the striatum; proteomic profiling revealed changes in proteins and pathways associated with metabolic, cellular organization, protein translation, and molecular transport processes. These findings are in contrast to the literature linking alcohol dependence to upregulation of neuroinflammatory processes, suggesting that the role of neuroinflammation in alcohol-related behavioral changes may vary across different brain regions. On the other hand, it is not clear whether the chronic drinking model employed induces alcohol dependence, and discrepancies with prior work could also be related to differences in the degree of alcohol exposure or the abstinence time point at which proteins were measured. Nonetheless, this work not only demonstrates the importance of subregion-specific characterization but also provides a new list of proteins that establishes a foundation for future investigations of alcohol-induced changes to striatal neuronal structure and cellular health and their behavioral consequences.

Semaglutide, a long-acting analogue of glucagon-like peptide-1, has emerged as an effective weight-loss treatment through appetite regulation in both diabetic and non-diabetic individuals. Accumulative evidence indicates its efficacy in weight loss-independent actions as well (Drucker, 2024). GLP-1 receptor agonists also have recently shown their potential as treatments for AUD in rodent and nonhuman primate studies, and clinical trials to evaluate the efficacy of the agonists have been initiated for AUD patients (Marty et al., 2020; Farokhnia et al., 2025; Hendershot et al., 2025). Aranas et al. examined, using rodent models in both male and female, the potential synergistic effects of combining semaglutide with the well-known anti-smoking agents, varenicline or bupropion, in the reduction of alcohol intake, while simultaneously assessing the impact of a high-fat diet (HFD) (Aranas et al.). Aranas et al. confirmed that semaglutide as a monotherapy effectively reduced alcohol intake and preference. Notably, when semaglutide was combined with either of varenicline, bupropion or HFD, it did not change the effects of semaglutide on the reduction of alcohol intake, suggesting that pharmacological interventions to target GLP-1 provide sufficient effects for AUD without requiring “complex combination regimens”, offering the potential for optimizing treatments for both AUD and obesity. Interestingly, HFD feeding in this study (Aranas et al.) was also found to be equally effective in reducing alcohol drinking, which is consistent with several rodent studies. Considering the nutritional deficiencies following prolonged chronic alcohol consumption and increased intake of palatable food in recovering patients, these data may have important clinical implications in the management of AUD. While a complex relationship between nutrition and AUD exists, the possibility of utilizing a non-pharmacological nutritional intervention that could facilitate the pathway of recovery needs further investigation (White and Sirohi, 2024).

Treatment availability for AUD continues to be limited, evident as less than 10% of patients obtain any form of treatment, and even the treatment rates vary around the world (Venegas et al., 2021). This gap in treatment has prompted exploration of alternative medications, particularly in regions where traditional practices are culturally relevant and accepted. For example, AUD and its comorbidities pose significant public health challenges in Africa, where traditional medicines are also suggested as a complementary and alternative option due to the limited availability of conventional medications. Maling et al. investigated the use of medicinal plants for treating AUD and its comorbidities by Traditional Medicine Practitioners (TMPs) in southwestern Uganda (Maling et al.). Using a descriptive cross-sectional ethnopharmacological survey, they documented diverse plant species employed by TMPs in the Bushenyi district for AUD treatment. While Maling et al. acknowledged potential bias from practitioners withholding sensitive plant information, this study indicates a significant level of agreement among TMPs regarding the use of the identified medicinal plants. Thus, this research serves dual purposes: highlighting the substantial gap in available treatments for AUD in Uganda while demonstrating valuable contribution of traditional medicine to addressing these conditions in Uganda. This provides important information about the use of culturally appropriate treatment approaches that could complement conventional therapies in resource-limited settings.

This research topic also covers an overview of potential therapeutic targets for AUD medications. *Fishler et al.* reviewed emerging pharmacological treatments for AUD (Fischler et al.), focusing on off-label medications including anticonvulsants, antipsychotics, antidepressants, and neuropeptides. Their analysis highlighted promising results for medications like topiramate, ondansetron, varenicline, neuropeptide Y, oxytocin, ghrelin inverse agonist, and gamma-hydroxybutyrate (GHB), while emphasizing that therapeutic standard implementation requires careful monitoring through comprehensive clinical trials. The review stressed that the comorbidity-reduction strategies would complement traditional treatments focusing on abstinence. It also emphasized that pharmacogenetic approaches may enhance treatment options and improve outcomes for AUD patients, potentially leading to optimized therapeutic effectiveness by enabling personalized medicine approaches.

In sum, this research topic aims to not only deepen our comprehension of AUD’s complex mechanisms but also provide updated information for advanced pharmacotherapeutic developments, providing additional help for individuals confronting this challenging condition. The neurobiological research suggests that alcohol elicits complex effects on proteomic profiles that vary across brain regions. This understanding opens new therapeutic targets focused on cellular organization and protein synthesis pathways. Pharmacological advances, particularly GLP-1 receptor agonists, and additional insights on possible nutritional interventions, such as HFD, offer promising prospects for expanding AUD treatment options. The documentation of traditional medicine practices also importantly contributes to our understanding of culturally accepted traditional treatment approaches that could be integrated with modern medical interventions. Future investigations should focus on translating this information into clinically viable treatments while maintaining awareness of practically available options that affect worldwide treatment implementation.
